# High Frequency of Symptomatic Zinc Deficiency in Infants in Northern Ethiopia

**DOI:** 10.1155/2014/719701

**Published:** 2014-12-07

**Authors:** Federica Dassoni, Zerihun Abebe, Federica Ricceri, Aldo Morrone, Cristiana Albertin, Bernard Naafs

**Affiliations:** ^1^Ayder Referral Hospital, Mekelle, Ethiopia; ^2^INMP Istituto Nazionale per la Promozione della Salute delle Popolazioni Migranti ed il Contrasto delle Malattie della Povertà, Via di San Gallicano 25, 00153 Roma, Italy; ^3^Unità Operativa di Dermatologia, Università di Milano, I.R.C.C.S. Fondazione Ca' Granda Ospedale Maggiore Policlinico, 20122 Milano, Italy; ^4^Dipartimento di Chirurgia e Medicina Traslazionale, Sezione di Dermatologia, Università degli Studi di Firenze, Viale Michelangelo 41, 50125 Firenze, Italy; ^5^Ospedale S. Camillo Forlanini, Piazza Carlo Forlanini 1, 00151 Rome, Italy; ^6^Dipartimento di Dermatologia, Università di Padova, Via Mocenigo 8, 35127 Padova, Italy; ^7^Stitching Tropen Dermatologie, Gracht 15, 8485KN15 Munnekeburen Friesland, The Netherlands

## Abstract

*Background*. Zinc deficiency occurs in infants when its demand exceeds its supply. It presents with cutaneous signs which, in severe cases, are associated with diarrhea, alopecia, and irritability. Genetic and acquired forms of zinc deficiency have been reported and often overlap clinical features. Malnutrition, prematurity, malabsorption syndromes, and burns may cause an increased demand for zinc. *Methods*. Cases of acquired transient infantile zinc deficiency (TIZD) observed during a period of 3 years at Ayder Referral Hospital of Mekelle, Northern Ethiopia, are reported here. Since no sophisticated tests were available at our center, the diagnosis was based on the clinical signs and prompt response to oral zinc supplementation. *Results*. We observed 18 cases of TIZD at our center. All patients were full-term and breastfeeding infants with no relevant associated diseases. *Conclusions*. In this region, a high incidence of this condition is observed. We could not rule out whether heterozygosity for the genetic mutation was present or that the disease was caused by a nutritional deficiency in the mothers or more probably because both the factors coexisted together. However, further studies are necessary to better understand the causes of the increased incidence of this disease in Northern Ethiopia.

## 1. Introduction

Zinc deficiency occurs in infants when its demand exceeds its supply. It presents with cutaneous signs which, in severe cases, are associated with diarrhea, alopecia, and irritability. Genetic and acquired forms of zinc deficiency have been described and often have overlapping clinical features. However, they usually differ in their time of presentation [1]. The genetic form of the disease, idiopathic acrodermatitis enteropathica (AE), is a rare autosomal recessive disease characterized by acral and periorificial dermatitis and low serum zinc levels [2]. The mutation occurs in the SLC39A4 gene, an intestinal zinc transporter. Since breast milk is thought to facilitate zinc absorption, it appears after the interruption of breast feeding and requires an unending zinc supplementation in most cases.

Transient infantile zinc deficiency (TIZD) is a disease clinically indistinguishable from idiopathic AE, though with different pathologic mechanisms. It occurs during the first 6 months of life, usually in infants with increased zinc requirements and/or inadequate diet concentrations of zinc. Malnutrition, prematurity, total parenteral nutrition, and burns may cause an increased demand for zinc. The supply of zinc to the growing child is reduced in congenital malabsorption syndromes. Nevertheless, zinc deficiency in healthy, full-term, breast-fed infants is also seen [3–11]. These deficiencies were related to low zinc levels in the maternal milk [12]. Heterozygosity for mutation of the gene SLC30A2 may be found in these cases [2–5]. This is known as transient neonatal zinc deficiency (TNZD).

Cutaneous lesions are observed in all the affected children, while more rarely they may be accompanied by diarrhea, irritability, alopecia, low grade fever, and conjunctivitis. Zinc deficiency is rapidly eliminated after treating the patients with oral zinc supplement, with prompt improvement in the clinical signs and symptoms.

The cases of transient infantile zinc deficiency (TIZD) observed during a 3-year period in Tigray region of Northern Ethiopia are reported here. Our observations indicate that this is a relatively frequent disease as compared with that in the other reports [8–14]. Diagnosis was based on clinical presentation and prompt response to oral zinc supplementation. To our knowledge, this is the first report on zinc deficiency encountered in patients at our center in Northern Ethiopia.

The aim of our report is to make the reader aware of the high presence of this rare and life threatening condition in Ethiopia, where it is often misdiagnosed by general doctors. It is therefore important to recognize and treat it properly. We hope further studies will lead to a better understanding of the causes of its high incidence.

## 2. Case Reports

We encountered 18 cases of symptomatic zinc deficiency at the Italian Dermatological Center of Ayder Hospital, Mekelle, Northern Ethiopia, from January 2008 to January 2011.

All the cases reported here showed typical clinical features of zinc deficiency of varying severity and duration. Patients were not severely malnourished and did not have evidence of growth retardation. One patient was moderately malnourished (underweight).

**Figure 1:**
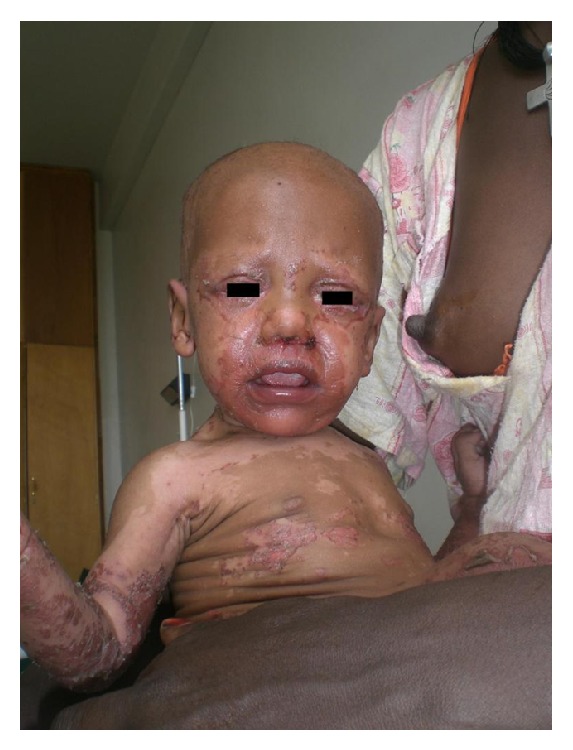
Periorificial lesions on face in an extensive case.

**Figure 2:**
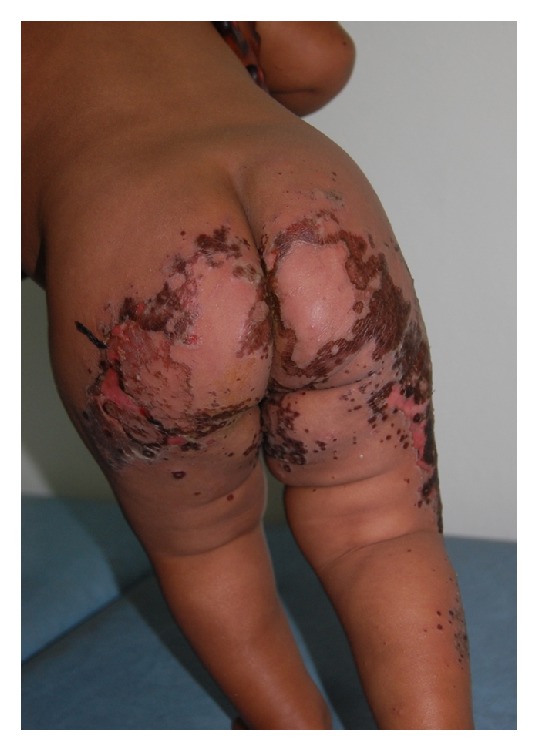
Lesions on buttocks. Marked margins, erosions, and brown crusts.

During a period of 3 years (January 2008 to January 2011), we encountered a total of 18 infants (11 females and 7 males, F : M = 1 : 0.6) aged 4 to 20 months with clinical skin features of acrodermatitis enteropathica. Lesions were symmetrical, well defined, erythematous, and often with ulcerations or erosions secondary to blisters, in some cases with overlying brown crusts. They were located on the periorificial areas (perianal, genital, nasal, ocular, and perioral), the limbs (mostly the lower limbs), the extremities, and in some cases the nape and the scalp (Figures 1 and 2).

One child presented with poliosis which underwent complete repigmentation after therapy with zinc supplement.

All children were totally or partially breast fed. Some of them were referred late to our facility by other health facilities after they had received systemic antibiotics without improvement with already widespread lesions. None of them had signs or symptoms of diarrhea, irritability, severe growth failure, or burns. Their general condition was good, except for that only child affected by moderate malnutrition.

Whether they were of normal birth weight and gestational age remained unknown. However, none of them reported any history of prematurity. Moreover, since they were living in rural villages with scanty health facilities, they were not likely to be significantly preterm unless they had a history of admission to the referral hospital.

Cutaneous manifestations were mostly moderate to severe and with ulceration or erosions (Table 1).

All of them showed a good and prompt improvement after short course of oral zinc supplement (3 mg/kg/day for 2-3 months). None of the patients had relapse of the lesions after discontinuing the treatment. For this reason, our diagnosis was transient neonatal zinc deficiency (TNZD).

Zinc levels in the patient's and mother's blood and in the mother's milk could not be measured, and mutation screening of the SLC30A2 gene could not be performed because of the lack of diagnostic facilities in this region of Ethiopia. However, all the mothers were in good general health and had no cutaneous manifestations.

One patient had positive family history for the same disease (one brother). We are not able to demonstrate whether other brothers/sisters presented mild signs of the disease; these were not reported by the mothers.

One patient presented with an unusual “facies” characterized by hypertelorism, prominent ears, and slightly small sized head, making us think of an associated chromosomal anomaly which was not possible to diagnose as there were no facilities for chromosome mapping.

One patient also had polydactyly, a common congenital defect encountered in this region.

One patient presented with associated scabies, which is also a highly prevalent disease in this region.

## 3. Discussion

Transient neonatal zinc deficiency (TNZD) is mainly observed in breast-fed infants and does not reoccur after weaning [4]. We think that the number of cases observed in Northern Ethiopia is very high as other reports in the literature are mostly single-case or two-case reports [2, 3, 6–12]. Taking into account the cases observed in full-term infants, to our knowledge only 15 cases have been reported since 1985 from different countries [1–3, 5–15]; it is therefore considered a rare disease.

Most of the previously reported cases in premature and also full-term infants were associated with low zinc levels in the maternal milk, although in some cases maternal zinc level was normal.

A low zinc level in the maternal milk is an important cofactor. Breast milk may be low in zinc because of a rare abnormality of zinc secretion by the mammary gland [16]. This may be the cause of symptomatic zinc deficiency, which is more severe and more common in premature infants because of the increased zinc requirements in this group. Symptomatic zinc deficiency can also appear from a combination of the SLC39A4 mutation in the infant and low milk zinc concentration from the mother who has the same heterozygous mutation [2].

All our patients had an excellent clinical improvement and discontinued the treatment after 2-3 months with no relapses. This indicates the diagnosis of TNZD and made us exclude AE, which requires lifelong treatment. Breast feeding, partial or total, was also a supporting factor for the diagnosis.

Zinc deficiency may also be secondary to a poor intestinal absorption or an increased urinary and intestinal secretion [13]. Disorders of intestinal malabsorption are other possible etiologic factors. None of the children reported here had clinical evidence of intestinal disease. It was not possible to measure urinary zinc levels. In our patients, we were not able to demonstrate any increased demand for zinc or any decreased ability of zinc storage. In fact, none of them was evidently preterm, had burns, had parenteral nutrition, or had any other evident reason to require increased zinc supplementation.

We could not rule out whether mother and child presented heterozygosity for a SLC39A4 or SLC30A2 gene mutation or whether the clinical features could be due to a dietary zinc deficiency of the mothers and/or increased zinc requirements of the infants. All the mothers were asymptomatic and had no skin abnormalities. As reported in previous studies from different regions of Ethiopia [17–19], they could also probably have a primary dietary asymptomatic zinc deficiency.

Since sophisticated diagnostic techniques are not generally available in developing countries, our diagnosis was clinical and confirmed by the prompt and remarkable healing of the lesions after treatment with oral zinc supplement. Most probably, an association of both heterozygosity for SLC30A2 gene mutation and dietary zinc deficiency in the mothers was contributing to the clinical manifestations in the infants.

Zinc is essential for growth, as it is involved in the development of the immune system, the muscles, and the bones, as well as the skin. In developing countries, diets often do not contain zinc in sufficient quantity or of sufficient bioavailability [18, 19]. Dietary zinc deficiency, as well as other nutritional deficiencies, has been reported from different regions of Ethiopia, affecting both pregnant women and children, although association with cutaneous signs has not been reported. Low levels of zinc in breast milk in Ethiopian mothers were reported in different studies [19–22], although there is no evidence of its association with clinical manifestations in children.

Most of the observed infants presented with signs of moderate or severe skin manifestations (14/18) and with ulcerations or erosions (14/18). This is probably due to the delayed access to our center. Beyond the difficult access to health facilities in rural areas of developing countries, some of our patients were previously treated at other health centers/hospitals with systemic or local antibiotics and only referred to us when no improvement was achieved.

Children were otherwise in good general health, except one case affected by moderate malnutrition.

We observed a preponderance of female infants affected (11 out of 18), although this is not statistically significant given the small number of patients. We could not identify a specific biological or cultural/behavioral reason which could explain the higher number of females affected.

Further studies are necessary to understand the causes of the increased incidence of TNZD in this population and to confirm the preponderance of female affected patients.

Transient neonatal zinc deficiency is a life threatening disease, often misdiagnosed by rural health workers and general doctors in Northern Ethiopia. Many of the reported patients were in fact in advanced stage conditions. If not diagnosed and treated properly, TNZD may have severe consequences on the child's growth. Keeping in mind the presence of the disease in the region is essential to recognize its clinical features and to give the correct treatment, as specific diagnostic tests are often not available in developing countries. Health workers should be made aware of the presence of the disease in order to refer to hospital all those patients who do not respond to first line therapy.

## Figures and Tables

**Table 1:** Mean features of the patients.

	Age (m)^*^	Sex	Severity of disease^**^	Ulcerations/erosions	Associated problems
1	5	F	Moderate	Yes	
2	7	F	Severe	Yes	White hair or poliosis
3	6	F	Mild/moderate	No	
4	4	M	Moderate	Yes	
5	8	M	Mild	Yes	Polydactyly
6	14	M	Moderate	Yes	
7	10	F	Severe and spread	No	
8	6	M	Moderate	Yes	Chromosomal anomaly
9	18	F	Moderate	Yes	
10	12	F	Moderate	Yes	Conjunctivitis, history of affected brother
11	16	F	Severe	Yes	Scabies, underweight
12	5	F	Severe	Yes	
13	8	F	Severe	Yes	
14	10	F	Mild	Yes	
15	5	F	Moderate	Yes	
16	20	M	Moderate	No	
17	4	M	Mild	Yes	
18	6	M	Moderate	No	

^*^(m): months. ^**^Mild: less than 10% BSA involved. Moderate: 10 to 30% BSA involved. Severe: more than 30% BSA involved. BSA = body surface area.
